# A timeline of freedom of movement in the European Economic Area

**DOI:** 10.12688/openreseurope.15042.1

**Published:** 2022-12-06

**Authors:** Emily Barker

**Affiliations:** 1 Social Statistics and Demography, University of Southampton, Southampton, SO17 1BJ, UK

**Keywords:** European Union, Migration, Freedom of Movement, EU Expansion, Single Market, European Economic Area, Migration Restrictions

## Abstract

The European Economic Area (EEA) provides a common market for goods, labour, services, and capital. Promoting integration between countries through the free movement of labour, or more generally persons, pre-dates the previous forms of the EEA. However, during the Southern and Eastern Expansions of the European Union, there have been transition agreements on persons, designed to restrict immigration. Opening up labour markets to the new member states with significantly lower GDP per capita than existing states, has been contentious. This is why the use of transition agreements have permitted periods which existing members can limit immigration. Not all existing member states impose restrictions, and during the Eastern Enlargements, the restrictions were imposed for varying lengths of time by different existing members up to a maximum of seven years. During the transition agreement, the economies of new members and existing members can converge, which is ultimately designed to limit the pull factor of migration. In this note, we provide a concise resource of the timeline of the expansion of full free movement of persons for countries in the EEA and Switzerland.

## 1 Introduction

The European Single Market includes the 27 countries of the European Union (EU) and the European Free Trade Association (EFTA) countries of Iceland, Liechtenstein, Norway, plus Switzerland. ^1^ Until 2020, the EU included the United Kingdom. The Single Market is for the free movement for goods, labour (alternatively persons), services, and capital. However, joining the Single Market has not always been as simple as joining and assuming equivalent status as Existing Member States (EMS). This prime example of international co-operation and international integration can come with caveats. Transition agreements on free movement of labour have been implemented on the countries joining the EU in 1981, 1986, 2004 (except Cyprus and Malta), 2007, and 2013. The transition agreements are in place to stop large shocks to the labour markets and population of EMS. Transition periods can last up to seven years, in which time it is probable that the economies of New Member States (NMS) have improved to be closer to EMS, and thus reducing the incentive to migrate. The expansion, exit of the UK, candidate and potential candidate countries of the EU is shown in Table 1.

**Table 1.** Expansion of the European Union.

Expansion of the EU								Brexit	CC	PCC
1958	1973	1981	1986	1995	2004	2007	2013	*2020*		
BEL	DNK	GRC	PRT	AUT	POL	BUL	HRV	-UK	ALB	BIH
FRA	IRE		ESP	FIN	CZE	ROU			MKD	KOS
DEU	UK			SWE	EST				MNE	
ITA					HUN				SRB	
LUX					LVA				TUR	
NED					LTU					
					SVK					
					SVN					
					CYP					
					MLT					

CC: candidate countries ; PCC: potential candidate countries. The development of the European Union and the possible future members. Source: European Union and European Commission

This paper provides researchers with a concise resource as to which years single market entry and freedom of movement was first obtained. Section 2 presents the expansion of the common market, and other relevant unions; Section 3 contains the years freedom of movement was gained; and Section 4 discusses possible future expansions and challenges.

## 2 Expansion of the Common Market

For each country we detail the year that they gained access to another country’s labour market. In finding these years, we have several policies to extract analysis from which we gather the joining dates between two (or more) countries. Below list the main treaties and evolution of the common (labour) market.

**European Coal and Steel Community (ECSC)** The founder members were Belgium, France, Italy, Luxembourg, the Netherlands, and West Germany. The Treaty establishing the European Coal and Steel Community Treaty came into force on 23rd July 1952. ^2^ This covered workers from only certain industries, thus not enabling full freedom of movement.

**European Economic Community (EEC)** The EEC succeeded the ECSC which aimed to establish a common market for the freedom of movement for goods, people, capital and services. This came into force 1st January 1958. Only by 1968 were any barriers to free movement of persons fully abolished, as preceding agreements still permitted countries to impose restrictions on foreign workers ( Condinanzi *et al.,* 2008).

**Treaty of Accession (ToA)** There were Treaties of Accession where new member countries joined the EU: 1972 for Denmark, Ireland and the United Kingdom, 1979 for Greece, 1985 for Spain and Portugal, and 1994 for Austria, Finland, and Sweden. The Treaties of Accession of 1979 and 1985 permitted transitional agreements which lasted until 1986 and 1992 respectively. There was not the expected large movements of people following during (or after) the transition period for Spain and Portugal, due to the improved economic (and political) conditions ( Royo, 2007), as such the transition period was reduced to six years. ^3^ The countries in the 2003 ToA included Cyprus, Czech Republic, Estonia, Latvia, Lithuania, Malta, Poland, Slovakia, and Slovenia. Citizens of Cyprus and Malta were allowed immediate access to all EU15 labour markets, but the remaining eight countries were not guaranteed this. Only Ireland, Sweden, and the United Kingdom fully opened their markets. Nations could impose restrictions on workers being able to access to the welfare state. The transition agreements permitted the NMS to employ reciprocal restrictions, which only Hungary, Poland and Slovenia did ( Goldner Lang, 2008).

**European Economic Area (EEA)** EEA includes the EU countries, Iceland, Liechtenstein, and Norway the agreement came into force on 1st January 1994. Austria, Finland and Sweden joined the EEA before subsequently joining the EU in 1995. The agreement brought the countries into the Single Market for the four freedoms. Not all of the EU policies were included in the agreement. EFTA today consists of Iceland, Liechtenstein, Norway, and Switzerland.

**Nordic Passport Union** A membership of Denmark, Finland, Iceland, Norway and Sweden made in 1954 enabling free movement between the nations with members implementing it at different dates.

**Switzerland** There are a number of safeguard agreements which applied to all countries when it came into force. Free movement in to Switzerland was suspended for a period under the safeguard agreement. The Eastern Expansion are subject to further delays on accessing the Swiss labour market beyond the seven years they are constrained to elsewhere in EU.

**Liechtenstein** The small country in the centre of Europe is an anomaly. A member of the EFTA, and a population of less than 40,000. ^4^ Working in the country is unrestricted for EEA and Swiss citizens but gaining a residence permit is more difficult due to the limitations allowed ( Cassis, 2012).

**The Withdrawal Agreement** In 2016, the United Kingdom voted to leave the EU. The terms of agreement were finalised in 2020. In the results, we have included the years which access were granted by the United Kingdom for access to its labour markets, and given to citizens of the United Kingdom in other European countries. Only citizens of the Republic of Ireland have free movement to the labour market of the United Kingdom and reciprocally to satisfy the Good Friday agreement.

**Figure 1.**
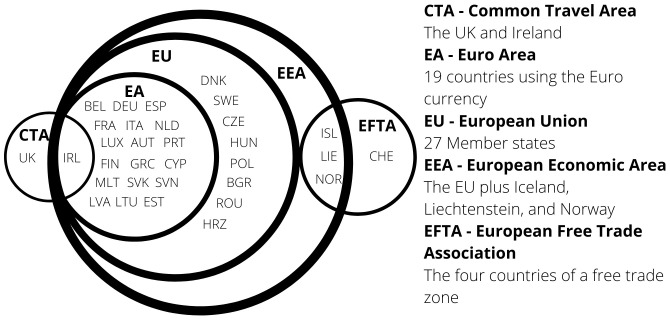
Groupings of Europe - 2021.

As a summary, Figure 1 shows the different economic groupings within Europe.

## 3 Results

Table 2 and Table 3 show the year in which a country gained full access to the labour market of another country. The column heading is the country that the row applies to. The row shows what year citizens of that country gained access to the labour market of the country in the column heading. For example, cell B4 of Table 2 shows that Bulgarian citizens gained full access to the labour market in 2014, whereas in D2 Austrian citizens were able to access the Bulgarian labour market in 2007 as no reciprocal measures were in place. 

**Table 2.** Expansion of Freedom of Movement (1).

Sending Country	Receiving Country															
	AUT	BEL	BGR	HRZ	CYP	CZE	DNK	EST	FIN	FRA	DEU	GRC	HUN	IRL	ITA	LVA
AUT	NA	1994	2007	2020	2004	2004	1994	2004	1994	1994	1994	1994	2009	1994	1994	2004
BEL	1994	NA	2007	2015	2004	2004	1973	2004	1994	1968	1968	1988	2009	1973	1968	2004
BGR	2014	2014	NA	2013	2007	2007	2009	2007	2007	2014	2014	2009	2009	2012	2012	2007
HRZ	2020	2015	2013	NA	2015	2013	2013	2013	2013	2015	2015	2015	2013	2013	2015	2013
CYP	2004	2004	2007	2015	NA	2004	2004	2004	2004	2004	2004	2004	2004	2004	2004	2004
CZE	2011	2009	2007	2013	2004	NA	2009	2004	2006	2008	2011	2006	2004	2004	2006	2004
DNK	1994	1973	2007	2013	2004	2004	NA	2004	1954	1973	1973	1988	2009	1973	1973	2004
EST	2011	2009	2007	2013	2004	2004	2009	NA	2006	2008	2011	2006	2004	2004	2006	2004
FIN	1994	1994	2007	2013	2004	2004	1954	2004	NA	1994	1994	1994	2006	1994	1994	2004
FRA	1994	1968	2007	2015	2004	2004	1973	2004	1994	NA	1968	1988	2008	1973	1968	2004
DEU	1994	1968	2007	2015	2004	2004	1973	2004	1994	1968	NA	1988	2009	1973	1968	2004
GRC	1994	1988	2007	2015	2004	2004	1988	2004	1994	1988	1988	NA	2006	1988	1988	2004
HUN	2011	2009	2007	2013	2004	2004	2009	2004	2006	2008	2011	2006	NA	2004	2006	2004
IRL	1994	1973	2007	2013	2004	2004	1973	2004	1994	1973	1973	1988	2004	NA	1973	2004
ITA	1994	1968	2007	2015	2004	2004	1973	2004	1994	1968	1968	1988	2006	1973	NA	2004
LVA	2011	2009	2007	2013	2004	2004	2009	2004	2006	2008	2011	2006	2004	2004	2006	NA
LTU	2011	2009	2007	2013	2004	2004	2009	2004	2006	2008	2011	2006	2004	2004	2006	2004
LUX	1994	1960	2007	2015	2004	2004	1973	2004	1994	1968	1968	1988	2007	1973	1968	2004
MLT	2004	2004	2007	2018	2004	2004	2004	2004	2004	2004	2004	2004	2004	2004	2004	2004
NLD	1994	1960	2007	2018	2004	2004	1973	2004	1994	1968	1968	1988	2007	1973	1968	2004
POL	2011	2009	2007	2013	2004	2004	2009	2004	2006	2008	2011	2006	2004	2004	2006	2004
PRT	1994	1992	2007	2013	2004	2004	1992	2004	1994	1992	1992	1992	2006	1992	1992	2004
ROU	2014	2014	2007	2013	2007	2007	2009	2007	2007	2014	2014	2009	2009	2012	2012	2007
SVK	2011	2009	2007	2013	2004	2004	2009	2004	2006	2008	2011	2006	2004	2004	2006	2004
SVN	2011	2009	2007	2018	2004	2004	2009	2004	2006	2008	2011	2006	2004	2004	2006	2004
ESP	1994	1992	2007	2015	2004	2004	1992	2004	1994	1992	1992	1992	2006	1992	1992	2004
SWE	1994	1994	2007	2013	2004	2004	1946	2004	1954	1994	1994	1994	2004	1994	1994	2004
ISL	1994	1994	2007	2015	2004	2004	1952	2004	1954	1994	1994	1994	2009	1994	1994	2004
LIE	1995	1995	2007	2018	2004	2004	1995	2004	1995	1995	1995	1995	2009	1995	1995	2004
NOR	1994	1994	2007	2014	2004	2004	1952	2004	1954	1994	1994	1994	2009	1994	1994	2004
CHE	2004	2004	2009	2024	2006	2006	2004	2006	2004	2004	2004	2004	2006	2004	2004	2006
UK	1994	1973	2007	2018	2004	2004	1973	2004	1994	1973	1973	1988	2004	1923	1973	2004

*Notes*: Years that free movement of persons was granted. The column shows the host country, with the row identifying the citizens of sending country. The UK ceased to be a member of the common labour market in 2020, though the original years are detailed here. Only Ireland and the UK have free movement.

**Table 3.** Expansion of Freedom of Movement (2).

Sending Country	Receiving Country															
	LTU	LUX	MLT	NLD	POL	PRT	ROU	SVK	SVN	ESP	SWE	ISL	LIE	NOR	CHE	UK
AUT	2004	1994	2004	1994	2007	1994	2007	2004	2006	1994	1994	1994	1995	1994	2007	1994
BEL	2004	1960	2004	1960	2007	1992	2007	2004	2006	1992	1994	1994	1995	1994	2007	1973
BGR	2007	2014	2014	2014	2007	2009	2007	2007	2007	2009	2007	2012	2012	2012	2016	2014
HRZ	2013	2015	2018	2018	2013	2013	2013	2013	2018	2015	2013	2015	2018	2014	2024	2018
CYP	2004	2004	2004	2004	2004	2004	2007	2004	2004	2004	2004	2004	2004	2004	2007	2004
CZE	2004	2007	2004	2007	2004	2006	2007	2004	2004	2006	2004	2009	2009	2009	2014	2004
DNK	2004	1973	2004	1973	2007	1992	2007	2004	2006	1992	1945	1955	1995	1954	2007	1973
EST	2004	2007	2004	2007	2004	2006	2007	2004	2004	2006	2004	2009	2009	2009	2014	2004
FIN	2004	1994	2004	1994	2006	1994	2007	2004	2006	1994	1949	1955	1995	1954	2007	1994
FRA	2004	1968	2004	1968	2007	1992	2007	2004	2006	1992	1994	1994	1995	1994	2007	1973
DEU	2004	1968	2004	1968	2007	1992	2007	2004	2006	1992	1994	1994	1995	1994	2007	1973
GRC	2004	1988	2004	1988	2006	1992	2007	2004	2006	1992	1994	1994	1995	1994	2007	1988
HUN	2004	2007	2004	2007	2004	2006	2007	2004	2004	2006	2004	2009	2009	2009	2014	2004
IRL	2004	1973	2004	1973	2004	1992	2007	2004	2004	1992	1994	1994	1995	1994	2007	1923
ITA	2004	1968	2004	1968	2006	1992	2007	2004	2006	1992	1994	1994	1995	1994	2007	1973
LVA	2004	2007	2004	2007	2004	2006	2007	2004	2004	2006	2004	2009	2009	2009	2014	2004
LTU	NA	2007	2004	2007	2004	2006	2007	2004	2004	2006	2004	2009	2009	2009	2014	2004
LUX	2004	NA	2004	1960	2007	1993	2007	2004	2006	1993	1994	1994	1995	1994	2007	1973
MLT	2004	2004	NA	2004	2004	2004	2007	2004	2004	2004	2004	2004	2004	2004	2007	2004
NLD	2004	1960	2004	NA	2007	1992	2007	2004	2006	1992	1994	1994	1995	1994	2007	1973
POL	2004	2007	2004	2007	NA	2006	2007	2004	2004	2006	2004	2009	2009	2009	2014	2004
PRT	2004	1993	2004	1992	2006	NA	2007	2004	2006	1992	1994	1994	1995	1994	2007	1992
ROU	2007	2014	2014	2014	2007	2009	NA	2007	2007	2009	2007	2012	2012	2012	2016	2014
SVK	2004	2007	2004	2007	2004	2006	2007	NA	2004	2006	2004	2009	2009	2009	2014	2004
SVN	2004	2007	2004	2007	2004	2006	2007	2004	NA	2006	2004	2009	2009	2009	2014	2004
ESP	2004	1993	2004	1992	2006	1992	2007	2004	2006	NA	1994	1994	1995	1994	2007	1992
SWE	2004	1994	2004	1994	2004	1994	2007	2004	2004	1994	NA	1955	1995	1954	2007	1994
ISL	2004	1994	2004	1994	2007	1994	2007	2004	2006	1994	1945	NA	1995	1954	2007	1994
LIE	2004	1995	2004	1995	2007	1995	2007	2004	2006	1995	1995	1995	NA	1995	2007	1995
NOR	2004	1994	2004	1994	2007	1994	2007	2004	2006	1994	1945	1955	1995	NA	2007	1994
CHE	2006	2004	2006	2004	2006	2004	2009	2006	2006	2004	2004	2004	2004	2004	NA	2004
UK	2004	1973	2004	1973	2004	1992	2007	2004	2004	1992	1994	1994	1995	1994	2007	NA

*Notes*: Years that free movement of persons was granted. The column shows the host country, with the row identifying the citizens of sending country. The UK ceased to be a member of the common labour market in 2020, though the original years are detailed here. Only Ireland and the UK have free movement.

### Notes

Spain allowed access to Bulgarian and Romanian citizens in 2009, but Spain reintroduced restrictions for Romanian citizens on 22nd July 2011, which were removed in 2014. Switzerland has a safeguard clause in their agreements, such that they are able to suspend free movement or introduce quotas on permits. They introduced quotas for category B permits in June 2013 for EU-15, Cyprus and Malta, and activated this safeguard clause in April 2012 for the EU- 8 countries ^5^ which were both removed in 2014. The original years for the United Kingdom remain as it is important to the history of the EU. The freedom of movement to and from the United Kingdom ends in 2020, *except* for Ireland.

## 4 Discussion

We have looked the evolution of the Single Market with a focus on the free movement of persons. This letter provides a resource for researchers looking at the history of the Europe and future paths. Briefly, we look at potential future expansions and the challenges.

### Future expansions of the EU

The CC and PCC listed in Table 1 have varying degrees of likelihood. Some of the countries have a significant length to go to so that their politics aligns with EU directives, and in some cases the country to be fully recognised as an independent state by all current member countries. Noteworthy examples including Cyprus, Greece, Romania, Slovakia, and Spain not recognising Kosovo; and the issues of Turkey and Cyprus over the Turkish Republic of Northern Cyprus, and absence of Turkish-Cypriot diplomatic relations. Expansion of the EU with CC and PCC is unlikely in the short-term, alongside opposition of founder EU members to further expansion as evidenced when a group of countries led by France blocked the opening talks with Albania and North Macedonia to the accession process in October 2019 citing the need for review and reform of the EU before any expansions can take place. ^6^

For any future (Eastern) expansions, transitional agreements on persons would likely be imposed. These agreements, designed to allow the closing the gap of NMS to EMS, are likely to be minimal due to the existing GDP per capita gap that exists. The real GDP per capita of Montenegro, North Macedonia, Serbia, Bosnia and Herzegovina, and Kosovo for 2019 was less than 25% of that of the EU-15, with Turkey at 37%. ^7^ The small closing of this gap will leave a pull factor to EMS, in particular the EU-14 and EFTA states, and possibly Slovenia. The inclusion of Slovenia towards EU-14 and EFTA states is due to their relatively high GDP per capita than other Eastern European countries, where wages and salaries are close to the levels of Greece, Italy, Portugal and Spain.

In addition, the fallout from Brexit within the United Kingdom cannot be ignored with support for independence in Scotland increasing, and the troubles associated with the Brexit agreement in Northern Ireland creating problems there. Scotland and Northern Ireland cannot be treated in the same way, with Northern Ireland’s requirements to satisfy the Good Friday agreement with the Republic of Ireland could see a reunified Ireland as one member of the EU, whereas Scotland would be an entirely separate state with no immediate right to be in the EU/EEA. ^8^

### Challenges of Integration

Countries joining the EU must align their social, economic and political factors with that of the EU. Further economic convergence is considered when a NMS joins the Euro currency. However, as shown in Figure 1, there are eight countries who have not adopted the Euro. Denmark negotiated an opt out and Sweden has no plans to, whilst the remaining six will join when they have met the necessary conditions. ^9^ From the perspective of a migrant, international migration is more challenging than domestic migration. The introduction of a common labour market has benefited millions of people, however, there are some issues that migrants encounter. One such struggle is a language barrier - there are 24 official languages of the EU with more languages in use in the common labour market such as Icelandic, Norwegian, and regional ones. Having a poor command of the host country’s language can be a barrier to employment or fully integrating into the community. Where a country has a positive attitude towards migrants, they are more likely to integrate ( Naveed & Wang, 2021), however, with the rise of populism in Western Europe in particular, negative attitudes are likely to increase.

## Data Availability

Zenodo: A timeline of freedom of movement in the European Economic Area. https://doi.org/10.5281/zenodo.7225880 ( Barker, 2022). This project contains the following underlying data: - Full list of sources by country Data are available under the terms of the Creative Commons Attribution 4.0 International license (CC-BY 4.0).

## References

[ref-6] Barker ER : A timeline of freedom of movement in the European Economic Area. Zenodo. 2022. 10.5281/zenodo.7225880 PMC10446036 37645342

[ref-1] Cassis I : Report: Free Movement of Workers, Report 1116899.European Free Trade Association.2012. Reference Source

[ref-2] Condinanzi M Lang A Nascimbene B : Citizenship of the Union and Freedom of Movement of Persons.Martinus Nijhoff Publishers, Leiden, The Netherlands.2008. Reference Source

[ref-3] Goldner Lang I : Transitional Arrangements in the Enlarged European Union: How Free is the Free Movement of Workers? *Croatian Yearbook of European Law and Policy.* 2008;3(3):241–271. 10.3935/cyelp.03.2007.35

[ref-4] Naveed A Wang C : Can Attitudes Toward Immigrant Explain Social Integration in Europe? EU versus Non-EU Migrant. *Social Indicator Research.* 2021;153:345–383. 10.1007/s11205-020-02492-8

[ref-5] Royo S : Lessons from Spain and Portugal in the European Union after 20 years. *Pôle Sud.* 2007;26:19–45. 10.3917/psud.026.0019

