# A timeline of freedom of movement in the European Economic Area

**DOI:** 10.12688/openreseurope.15042.3

**Published:** 2024-03-21

**Authors:** Emily Barker

**Affiliations:** 1 Social Statistics and Demography, University of Southampton, Southampton, SO17 1BJ, UK

**Keywords:** European Union, Migration, Freedom of Movement, EU Expansion, Single Market, European Economic Area, Migration Restrictions

## Abstract

The European Economic Area (EEA) provides a common market for goods, labour, services, and capital. Promoting integration between countries through the free movement of labour, or more generally persons, pre-dates the previous forms of the EEA. However, during the Southern and Eastern Expansions of the European Union, there have been transition agreements on persons, designed to restrict immigration. Opening up labour markets to the new member states with significantly lower GDP per capita than existing states, has been contentious. This is why the use of transition agreements have permitted periods which existing members can limit immigration. Not all existing member states impose restrictions, and during the Eastern Enlargements, the restrictions were imposed for varying lengths of time by different existing members up to a maximum of seven years. During the transition agreement, the economies of new members and existing members can converge, which is ultimately designed to limit the pull factor of migration. In this note, we provide a concise resource of the timeline of the expansion of full free movement of persons for countries in the EEA and Switzerland.

## Plain language summary

In this research we answer: What year were citizens of the countries within the European Economic Area (EEA) able to move to another country in the EEA without any restrictions? Immigration is a controversial topic in most developed economies, but the EEA is a unique group of countries which offers its citizens the ability to live and work anywhere in its member countries. Political issues have risen when there are new members who have significantly lower GDP per capita than existing member states. To satisfy some of the existing members, they can limit migration from the new members for up to seven years. This research brings together many sources to detail when restrictions were lifted if this differed to the country joining the EU or EEA.

## 1 Introduction

The European Single Market includes the 27 countries of the European Union (EU) and the European Free Trade Association (EFTA) countries of Iceland, Liechtenstein, Norway, plus Switzerland. ^1^ Until 2020, the EU included the UK. The Single Market promotes the free movement for goods, labour (alternatively persons), services, and capital. However, joining the Single Market has not always been as simple as joining and gaining immediate access to all forms of free movement, especially labour, as existing member states (EMS) have. This prime example of international co-operation and international integration can come with caveats. Transition agreements on free movement of labour have been implemented on the countries joining the EU in 1981, 1986, 2004 (except Cyprus and Malta), 2007, and 2013. The transition agreements are in place to stop large shocks to the labour markets and population of EMS. The transition periods can last up to seven years, in which time it is probable that the economies of new member states (NMS) have improved to be closer to EMS. The restrictions on free movement of labour were in conventional sense only, which enabled exceptions and ways to work around the restrictions. As a result of restrictions, there should be reduced incentive to migrate. The 15 members that joined the EU before 2004 are commonly referred to as the EU15. The expansion, exit of the UK, candidate and potential candidate countries of the EU is shown in Table 1.

**Table 1.** Expansion of the European Union.

		Expansion of the EU						Brexit	CC	PCC
1958	1973	1981	1986	1995	2004	2007	2013	*2020*		
BEL	DNK	GRC	PRT	AUT	POL	BUL	HRV	-UK	ALB	KOS
FRA	IRE		ESP	FIN	CZE	ROU			BIH	
DEU	UK			SWE	EST				GEO	
ITA					HUN				MKD	
LUX					LVA				MDA	
NED					LTU				MNE	
					SVK				SRB	
					SVN				TUR	
					CYP				UKR	
					MLT					

CC: candidate countries; PCC: potential candidate countries. The development of the European Union and the possible future members. In June 2022, Moldova and Ukraine were granted Candidate Country status, Bosnia and Herzegovina in December 2022, and Georgia in December 2023. Correct as of March 2024. Source: European Union and European Commission

This paper provides a concise resource as to which years single market entry and freedom of movement was first obtained to enable researchers to easily access the information. Section 2 describes the methodology employed; Section 3 presents the expansion of the common market, and other relevant unions; and contains the years freedom of movement was gained; and Section 4 discusses possible future expansions and challenges.

## 2 Methodology

### Study design

The most suitable approach for this research is a document analysis of the 32 countries of the EU+ in 2019. Further, in the Eastern Expansions since 2004, there has been differing results across the EMS. The primary method used is document analysis - the exact sources are detailed in the next sections. To formulate a timeline of freedom of movement, we proceed with two main questions: (i) what date did a country join the common market and (ii) was that country granted immediate freedom of movement for persons. If the answer for the second question is negative, we are required to explore further sources to find the years which full access was granted. Early research stages required creating a timeline of the evolution of the EEA (as detailed in Section 3). It is important to note that the restrictions on free movement of labour were on workers specifically and not all types of migration. As services are not subject to restrictions, it is possible for self-employed workers to move or establish themselves while their home country is under transition restrictions. A founding legal case from 1990 brought by a Portuguese company to the European courts determined that the Portuguese company was entitled to bring its own workers to France to complete the works that the firm had been contracted to do, instead of hiring French workers ( European Court, 1991; Marshall, 1991). This was at a time when Portugal was under a transition agreement. The ability for self-employed Polish workers, even doctors, to work in Germany is discussed in Fellmer (2008); the process was replicated across countries that imposed restrictions.

### Source selection

To gather information for the timeline on the expansion of the European Common Labour Market, we begin by researching the timeline of what is currently known as the EEA. The EU provides a record of legal agreements on EUR-Lex which the majority of treaties included in this research are available. For information not available from EUR-Lex, we source from other government sources, academic literature or reports from official organisations.

### Data collection

The analysis of the downloaded documents took place in July and August 2021. The conclusion of the research occurred when Table 2 and Table 3 were complete. The full list of sources by country is available in the data accompanying this research ( Barker, 2022). The treaties covered most of the details, however, some were details that could be subject to change. ^2^ The treaties were chosen as they are the legal documents and available from credible sources. Important treaties included in this investigation were the Benelux Economic Union Benelux Union (1958); the Treaty establishing the ECSC Publications Office of the European Union (1951); the Treaty establishing the EEC The six Member States: Belgium, Germany, France, Italy, Luxembourg, Netherlands (1957); Treaties of Accessions European Communities (1972); European Communities (1979); European Communities (1985); European Communities (1994); European Union (2003); European Union (2005); European Union (2012); the establishment of the EEA Council of the European Union, European Commission (1993); and the withdrawal of the UK European Union (2020).

**Table 2.** Expansion of Freedom of Movement (1).

Sending Country	Receiving Country															
	AUT	BEL	BGR	HRV	CYP	CZE	DNK	EST	FIN	FRA	DEU	GRC	HUN	IRL	ITA	LVA
AUT		1994	2007	2020	2004	2004	1994	2004	1994	1994	1994	1994	2009	1994	1994	2004
BEL	1994		2007	2015	2004	2004	1973	2004	1994	1968	1968	1988	2009	1973	1968	2004
BGR	2014	2014		2013	2007	2007	2009	2007	2007	2014	2014	2009	2009	2012	2012	2007
HRV	2020	2015	2013		2015	2013	2013	2013	2013	2015	2015	2015	2013	2013	2015	2013
CYP	2004	2004	2007	2015		2004	2004	2004	2004	2004	2004	2004	2004	2004	2004	2004
CZE	2011	2009	2007	2013	2004		2009	2004	2006	2008	2011	2006	2004	2004	2006	2004
DNK	1994	1973	2007	2013	2004	2004		2004	1954	1973	1973	1988	2009	1973	1973	2004
EST	2011	2009	2007	2013	2004	2004	2009		2006	2008	2011	2006	2004	2004	2006	2004
FIN	1994	1994	2007	2013	2004	2004	1954	2004		1994	1994	1994	2006	1994	1994	2004
FRA	1994	1968	2007	2015	2004	2004	1973	2004	1994		1968	1988	2008	1973	1968	2004
DEU	1994	1968	2007	2015	2004	2004	1973	2004	1994	1968		1988	2009	1973	1968	2004
GRC	1994	1988	2007	2015	2004	2004	1988	2004	1994	1988	1988		2006	1988	1988	2004
HUN	2011	2009	2007	2013	2004	2004	2009	2004	2006	2008	2011	2006		2004	2006	2004
IRL	1994	1973	2007	2013	2004	2004	1973	2004	1994	1973	1973	1988	2004		1973	2004
ITA	1994	1968	2007	2015	2004	2004	1973	2004	1994	1968	1968	1988	2006	1973		2004
LVA	2011	2009	2007	2013	2004	2004	2009	2004	2006	2008	2011	2006	2004	2004	2006	
LTU	2011	2009	2007	2013	2004	2004	2009	2004	2006	2008	2011	2006	2004	2004	2006	2004
LUX	1994	1960	2007	2015	2004	2004	1973	2004	1994	1968	1968	1988	2007	1973	1968	2004
MLT	2004	2004	2007	2018	2004	2004	2004	2004	2004	2004	2004	2004	2004	2004	2004	2004
NLD	1994	1960	2007	2018	2004	2004	1973	2004	1994	1968	1968	1988	2007	1973	1968	2004
POL	2011	2009	2007	2013	2004	2004	2009	2004	2006	2008	2011	2006	2004	2004	2006	2004
PRT	1994	1992	2007	2013	2004	2004	1992	2004	1994	1992	1992	1992	2006	1992	1992	2004
ROU	2014	2014	2007	2013	2007	2007	2009	2007	2007	2014	2014	2009	2009	2012	2012	2007
SVK	2011	2009	2007	2013	2004	2004	2009	2004	2006	2008	2011	2006	2004	2004	2006	2004
SVN	2011	2009	2007	2018	2004	2004	2009	2004	2006	2008	2011	2006	2004	2004	2006	2004
ESP	1994	1992	2007	2015	2004	2004	1992	2004	1994	1992	1992	1992	2006	1992	1992	2004
SWE	1994	1994	2007	2013	2004	2004	1946	2004	1954	1994	1994	1994	2004	1994	1994	2004
ISL	1994	1994	2007	2015	2004	2004	1952	2004	1954	1994	1994	1994	2009	1994	1994	2004
LIE	1995	1995	2007	2018	2004	2004	1995	2004	1995	1995	1995	1995	2009	1995	1995	2004
NOR	1994	1994	2007	2014	2004	2004	1952	2004	1954	1994	1994	1994	2009	1994	1994	2004
CHE	2004	2004	2009	2022	2006	2006	2004	2006	2004	2004	2004	2004	2006	2004	2004	2006
UK	1994	1973	2007	2018	2004	2004	1973	2004	1994	1973	1973	1988	2004	1923	1973	2004

*Notes:* Years that free movement of persons was first granted. The column shows the host country, with the row identifying the citizens of sending country. The UK ceased to be a member of the common labour market in 2020, though the original years are detailed here. Only Ireland and the UK have free movement.

**Table 3.** Expansion of Freedom of Movement (2).

Sending Country	Receiving Country															
	LTU	LUX	MLT	NLD	POL	PRT	ROU	SVK	SVN	ESP	SWE	ISL	LIE	NOR	CHE	UK
AUT	2004	1994	2004	1994	2007	1994	2007	2004	2006	1994	1994	1994	1995	1994	2007	1994
BEL	2004	1960	2004	1960	2007	1992	2007	2004	2006	1992	1994	1994	1995	1994	2007	1973
BGR	2007	2014	2014	2014	2007	2009	2007	2007	2007	2009	2007	2012	2012	2012	2016	2014
HRV	2013	2015	2018	2018	2013	2013	2013	2013	2018	2015	2013	2015	2018	2014	2022	2018
CYP	2004	2004	2004	2004	2004	2004	2007	2004	2004	2004	2004	2004	2004	2004	2007	2004
CZE	2004	2007	2004	2007	2004	2006	2007	2004	2004	2006	2004	2009	2009	2009	2011	2004
DNK	2004	1973	2004	1973	2007	1992	2007	2004	2006	1992	1945	1955	1995	1954	2007	1973
EST	2004	2007	2004	2007	2004	2006	2007	2004	2004	2006	2004	2009	2009	2009	2011	2004
FIN	2004	1994	2004	1994	2006	1994	2007	2004	2006	1994	1949	1955	1995	1954	2007	1994
FRA	2004	1968	2004	1968	2007	1992	2007	2004	2006	1992	1994	1994	1995	1994	2007	1973
DEU	2004	1968	2004	1968	2007	1992	2007	2004	2006	1992	1994	1994	1995	1994	2007	1973
GRC	2004	1988	2004	1988	2006	1992	2007	2004	2006	1992	1994	1994	1995	1994	2007	1988
HUN	2004	2007	2004	2007	2004	2006	2007	2004	2004	2006	2004	2009	2009	2009	2011	2004
IRL	2004	1973	2004	1973	2004	1992	2007	2004	2004	1992	1994	1994	1995	1994	2007	1923
ITA	2004	1968	2004	1968	2006	1992	2007	2004	2006	1992	1994	1994	1995	1994	2007	1973
LVA	2004	2007	2004	2007	2004	2006	2007	2004	2004	2006	2004	2009	2009	2009	2011	2004
LTU		2007	2004	2007	2004	2006	2007	2004	2004	2006	2004	2009	2009	2009	2011	2004
LUX	2004		2004	1960	2007	1993	2007	2004	2006	1993	1994	1994	1995	1994	2007	1973
MLT	2004	2004		2004	2004	2004	2007	2004	2004	2004	2004	2004	2004	2004	2007	2004
NLD	2004	1960	2004		2007	1992	2007	2004	2006	1992	1994	1994	1995	1994	2007	1973
POL	2004	2007	2004	2007		2006	2007	2004	2004	2006	2004	2009	2009	2009	2011	2004
PRT	2004	1993	2004	1992	2006		2007	2004	2006	1992	1994	1994	1995	1994	2007	1992
ROU	2007	2014	2014	2014	2007	2009		2007	2007	2009	2007	2012	2012	2012	2016	2014
SVK	2004	2007	2004	2007	2004	2006	2007		2004	2006	2004	2009	2009	2009	2011	2004
SVN	2004	2007	2004	2007	2004	2006	2007	2004		2006	2004	2009	2009	2009	2011	2004
ESP	2004	1993	2004	1992	2006	1992	2007	2004	2006		1994	1994	1995	1994	2007	1992
SWE	2004	1994	2004	1994	2004	1994	2007	2004	2004	1994		1955	1995	1954	2007	1994
ISL	2004	1994	2004	1994	2007	1994	2007	2004	2006	1994	1945		1995	1954	2007	1994
LIE	2004	1995	2004	1995	2007	1995	2007	2004	2006	1995	1995	1995		1995	2007	1995
NOR	2004	1994	2004	1994	2007	1994	2007	2004	2006	1994	1945	1955	1995		2007	1994
CHE	2006	2004	2006	2004	2006	2004	2009	2006	2006	2004	2004	2004	2004	2004		2004
UK	2004	1973	2004	1973	2004	1992	2007	2004	2004	1992	1994	1994	1995	1994	2007	

*Notes:* Years that free movement of persons was first granted and for Switzerland the year that free movement with quotas was granted. The column shows the host country, with the row identifying the citizens of sending country. The UK ceased to be a member of the common labour market in 2020, though the original years are detailed here. Only Ireland and the UK have free movement.

### Analysis

The data analysis method employed is basic quantitative content analysis, in which we search the required documents for the dates to answer the questions outlined in our study design. The dates gathered from this research are used to create Table 2 and Table 3. In addition, the exact dates (not only years) Barker and Bijak (2021) used to create a variable for the effective labour market size for EEA states and Switzerland for the purpose of the investigation of the effects of net immigration or net emigration on the macroeconomy. As there has been considerable expansion of the EEA since the start of the sampling period (2002), this needed to be reflected as an exogenous variable in that model. It was important to identify the dates that countries joined the bloc (and exited in the case of the UK) to reflect the joining of new members and their labour force size. For example, when the A8 countries joined in 2004, there was a significant increase in migration to Ireland, Sweden and the UK because those countries did not place any limitations on movements. The other member states did impose limits, but for varying lengths of time which meant reflecting the changes was important. While it was necessary to the research to find the changes from 2002, to complete the research we backdated the start of the study to have an understanding of the origins and development of the European Common Market as of today.

## 3 Results

### Expansion of the common market

For each country we detail the year that they gained access to another country’s labour market. In finding these years, we have several policies to extract analysis from which we gather the joining dates between two (or more) countries. Below we list the main treaties and evolution of the common (labour) market. The agreements are signed and agreed by all the EMS and NMS up to years in advance of the NMS or changes occurring, by which time these are enshrined in national law.

**European Coal and Steel Community (ECSC)** The founder members were Belgium, France, Italy, Luxembourg, the Netherlands, and West Germany. The Treaty establishing the European Coal and Steel Community Treaty came into force on 23rd July 1952 ( Publications Office of the European Union, 1951). This covered workers from only certain industries, thus not enabling full freedom of movement.

**European Economic Community (EEC)** The EEC succeeded the ECSC which aimed to establish a common market for the freedom of movement for goods, people, capital and services. This came into force 1st January 1958. Only by 1968 were any barriers to free movement of persons fully abolished, as preceding agreements still permitted countries to impose restrictions on foreign workers ( Condinanzi *et al.*, 2008).

**Treaty of Accession** There were Treaties of Accession where new member countries joined the EU: 1972 for Denmark, Ireland and the UK to join in 1973; 1979 for Greece to join in 1981; 1985 for Spain and Portugal to join in 1986; 1994 for Austria, Finland, and Sweden to join in 1995; 2003 for 10 countries to join in 2004; 2005 for Bulgaria and Romania to join in 2007, and 2011 for Croatia to join in 2013. The Treaties of Accession of 1979 and 1985 permitted transitional agreements which lasted until 1986 and 1992 respectively. There was not the expected large movements of people following during (or after) the transition period for Spain and Portugal, due to the improved economic (and political) conditions ( Royo, 2007), as such the transition period was reduced to six years ( Council of the European Union, 1991). The countries in the 2003 Treaty included Cyprus, Czech Republic, Estonia, Latvia, Lithuania, Malta, Poland, Slovakia, and Slovenia. Citizens of Cyprus and Malta were allowed immediate access to all the EU15 and EFTA (except Switzerland) labour markets, but the remaining eight countries were not guaranteed this. Only Ireland, Sweden, and the UK fully opened their markets. Nations could impose restrictions on workers being able to access the welfare state. The 2005 Treaty included Bulgaria and Romania, for both countries there were restrictions on freedom of movement from most of the same countries as before, plus Hungary Ireland, Malta and the UK. The 2011 Treaty covered Croatia’s accession which again featured restrictions on full freedom of movement. The transition agreements permitted the NMS to employ reciprocal restrictions, which only Hungary, Poland and Slovenia of the A8 countries did ( Goldner Lang, 2008) and latterly Croatia. Croatia was stricter about the reciprocal restrictions as the three A8 countries lifted them all by 2009 at the latest, even though Austria and Germany stilled imposed the restrictions until 2011.

**European Economic Area (EEA)** EEA includes the EU countries, Iceland, Liechtenstein, and Norway the agreement came into force on 1st January 1994. Austria, Finland and Sweden joined the EEA before subsequently joining the EU in 1995. The agreement brought the countries into the Single Market for the four freedoms. Not all of the EU policies were included in the agreement. EFTA today consists of Iceland, Liechtenstein, Norway, and Switzerland.

**Nordic Passport Union** A membership of Denmark, Finland, Iceland, Norway and Sweden made in 1954 enabling free movement between the nations with members implementing it at different dates.

**Switzerland** Switzerland’s freedom of movement is unlike any other member. They are not a member of the EEA so do not abide by those rules. Instead, Switzerland and the EU have agreements in place. With regards to freedom of movement of persons, this began with the Agreement on the Free Movement of Persons (AFMP) ( European Communities, 1999). The AFMP lifts restrictions on EU citizens wishing to live or work in Switzerland. It was signed in 1999 and came into force in June 2002 ^3^. There are a number of safeguard agreements which applied to all countries when respective country groupings came into force. There are different levels of freedom of movement of labour (and persons), including Swiss national worker priority, quotas, free movement of persons with and without safeguard limits, and full freedom without limitations. As detailed by the State Secretariat for Migration (2023), for the first two years of the agreement, the EU15 countries and EFTA members were under national worker priority, with quotas between 2004 and 2007. Cyprus and Malta were under the quota scheme for 2006–2007. Free movement with safeguard clauses lasted until 2013, when the safeguard clause was activated for a year, and since deactivation, there has been full freedom of movement. The Eastern Expansions are subject to further delays on accessing the Swiss labour market. The A8 countries experienced national worker priority from 2006 to 2011, when free movement with clauses was attained for one year, until the invocation of the clause from 2012–2014. Since 2014, there has been free movement of persons without limitations. Bulgaria and Romania were subject to national worker priority from 2009 to 2016, one year of safeguard clauses before two years where the invocation of a safeguard clause. This was lifted in 2019, with full freedom of movement since. Croatia experienced country specific quotas from 2014 to the end of 2016, from 2017 to the end of 2021, there was national worker priority. Switzerland applied the permit system for Croatia, starting in 2022, on a trial basis. In 2022 and 2023, the immigration of Croatian nationals was at a level that the safeguard clause was activated to impose quotas for 2023 and 2024 ^4^. The plan is to lift the quota for 2025 and 2026 before allowing freedoms without limitations in 2027. To be consistent across countries, and the definition in European Communities (1999), we use the free movement with safeguard clauses in our research.

**Liechtenstein** The small country, or micro-state, in the centre of Europe is a unique case. A member of the EFTA in its own right, and a population of less than 40,000. ^5^ Working in the country is unrestricted for EEA and Swiss citizens but gaining a residence permit is more difficult due to the limitations allowed ( Cassis, 2012). Countries of a similar size, e.g., Andorra, Monaco, San Marino, and the Vatican City have agreements with the EU to be de facto members ^6^ as well as using the Euro currency, while Liechtenstein uses the Swiss Franc.

**The Withdrawal Agreement** In 2016, the UK voted to leave the EU. The terms of agreement were finalised in 2020. In the results, we have included the years which access were granted by the UK to its labour markets and given to citizens of the UK in other European countries. Only citizens of the Republic of Ireland have free movement to the labour market of the UK and reciprocally to satisfy the Good Friday Agreement.

**Figure 1.**
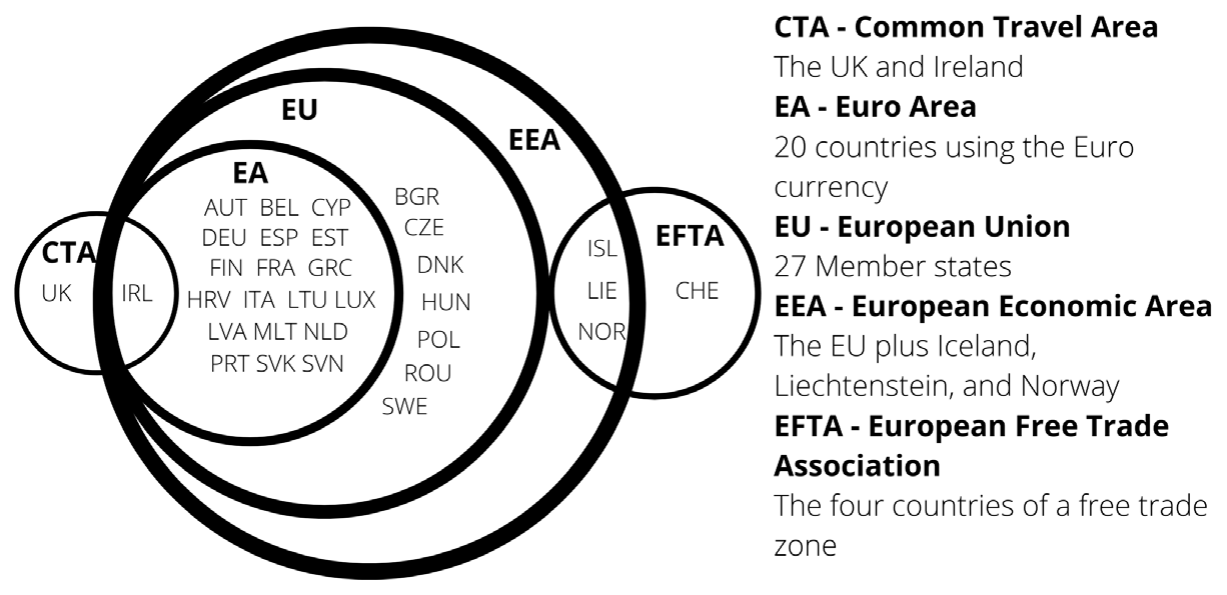
Groupings of Europe - 2023.

As a summary, Figure 1 shows the different economic groupings within Europe. The most recent county to join the Euro Area is Croatia, which changed over in 2023. The next members to join are likely to be Bulgaria and Czechia ^7^.

Table 2 and Table 3 show the year in which a country gained full access to the labour market of another country. The column heading is the country that the row applies to. The row shows what year citizens of that country gained access to the labour market of the country in the column heading. For example, cell B4 of Table 2 shows that Bulgarian citizens gained full access to the labour market in 2014, whereas in D2 Austrian citizens were able to access the Bulgarian labour market in 2007 as no reciprocal measures were in place. These are the original years that there was access, however, there are some changes where freedom was temporarily revoked as detailed in the section covering Switzerland and below.

### Notes

Spain allowed access to Bulgarian and Romanian citizens in 2009, but Spain reintroduced restrictions for Romanian citizens on 22 July 2011, which were removed in 2014. Switzerland has a safeguard clause in their agreements, such that they are able to suspend free movement or introduce quotas on permits. The original years for the UK remain as it is important to the history of the EU. The freedom of movement to and from the UK ends in 2020, *except* for Ireland.

## 4 Discussion

We have looked the evolution of the Single Market with a focus on the free movement of persons. This case study provides a resource for researchers looking at the history of the European common (labour) market, and future paths with respect to potential restrictions for future expansions. In light of the results presented in Table 2 and Table 3, it is clear that simply assuming that freedom of movement of workers and persons in the conventional sense was granted in the years of accession is incorrect. Only in the case of the founding members of the EEC and the 1972 and 1994 accession countries was this correct. For researchers studying migration in Europe, and to the wider European labour market, this gives a timeline of major changes. This is not to say that all future expansions will include restrictions, though as the next subsections will consider, this explains some foundations and likelihoods of expansion restrictions. When future expansions of the EU happen, researchers will be able to use this concise resource to see how previous expansions dealt with the restrictions on freedom of movement. In this section, we look at assessing the effectiveness of expansions, the potential future expansions and the challenges of integration.

### Assessing the effectiveness

Future potential members of the EU must consider whether joining the EU is of net benefit to the country, while the EU14 ^8^ must evaluate the future of the EU in terms of potential reforms ( Costa *et al.*, 2023). Nevertheless, has joining the common market been beneficial? Macroeconomic based studies have shown that membership for the Central and Eastern European countries has beneficial as these countries have experienced economic convergence relative to the EU15 and accelerated economic growth since joining the common market driven by increased trade, foreign investment and government integrity ( Nagy & Šiljak, 2022; Rapacki & Prochniak, 2019). The effects of labour are not studied to the same degree. To empirically analyse the effectiveness of the freedom of movement for labour on individual member states would be possible with extensive review of labour market data. This would highlight whether problems in the labour market, including (industry specific) labour shortages, have benefited from common market membership. There are two distinct groups within the common labour market: the net senders of migrants and net receivers of migrants. Migration is a contentious topic, particularly in the countries that have high net migration rates which has led to the aversion of further expansions as mentioned previously. The question that these countries must ask can be likened to Switzerland’s position when negotiating the bilateral agreements: does the (perceived) trade-off for access to the common market for goods, services and capital exceed that for high levels of immigration? A conclusion from Switzerland can be seen that the trade-off is worth it. Though, if we consider the case of the UK, the Government pursued a complete exit from the common market. Opinion polls have seen a trend of increase to the opinion that the UK was wrong to leave the EU. It is important to note that Switzerland regularly has referendums, while the UK only had one vote in 2016 and none to accept or reject the agreed deal. Both examples suggest that the trade-off is perceived to be worthwhile since recent polling has been done since the effects of the deal have become reality. From the opposing side, the candidate countries are all likely to face emigration if they were to join, as discussed above. Nevertheless, their governments see access to the single market beneficial even if there is large emigration.

### Future expansions of the EU

The candidate countries and potential candidate countries listed in Table 1 have varying degrees of likelihood. Some of the countries have a significant length to go to so that their politics aligns with EU directives, and in some cases the country to be fully recognised as an independent state by all current member countries. Noteworthy examples include Cyprus, Greece, Romania, Slovakia, and Spain not recognising Kosovo; and the issues of Turkey and Cyprus over the Turkish Republic of Northern Cyprus, and absence of Turkish-Cypriot diplomatic relations though negotiations with Turkey have been frozen ^9^ Expansion of the EU is unlikely in the short-term due to the status of negotiations with each candidate country only in early stages of negotiations ^10^, alongside opposition of founder EU members to further expansion as evidenced when a group of countries led by France blocked the opening talks with Albania and North Macedonia to the accession process in October 2019 citing the need for review and reform of the EU before any expansions can take place. ^11^

For any future (Eastern) expansions, transitional agreements on persons would likely be imposed. These agreements, designed to allow the closing of the gap of NMS to EMS, are likely to be minimal due to the existing GDP per capita gap that exists. The real GDP per capita of Montenegro, North Macedonia, Serbia, Bosnia and Herzegovina, and Kosovo for 2019 was less than 25% of that of the EU-15, with Turkey at 37%. ^12^ The small closing of this gap will leave a pull factor to EMS, in particular the EU-14 and EFTA states, and possibly Slovenia. The inclusion of Slovenia towards EU-14 and EFTA states is due to their relatively high GDP per capita than other Eastern European countries, where wages and salaries are close to the levels of Greece, Italy, Portugal and Spain.

In addition, the fallout from Brexit within the UK raised questions over Northern Ireland and Scotland’s membership ambitions. For Northern Ireland, there have been troubles associated with the Brexit agreement and Northern Ireland’s requirements to satisfy the Good Friday agreement with the Republic of Ireland. In theory, there could be a reunified Ireland as one member of the EU. The Scottish National Party (SNP) asked for a second referendum to be held as recently as 2022, however, this was ultimately rejected by the UK Government, and soon after the UK Supreme Court ruled that a referendum cannot be held without UK Government approval. ^13^ Scotland voted to remain in the EU in the Brexit referendum, but Scotland would be an entirely separate state with no immediate right to be in the EU/EEA. ^14^

### Challenges of integration

With the recent rise in candidate countries, and belief that expansion of the EU is inevitable, it is important to consider problems that countries face during transitional stages from candidate country to full members. Countries joining the EU must align their social, economic and political status and beliefs with that of the EU, in particular democracy, human rights, and international cooperation. Further economic convergence is considered when a NMS joins the Euro currency. However, as shown in Figure 1, there are seven countries who have not adopted the Euro. Denmark negotiated an opt out and Sweden has no plans to, whilst the remaining five will join when they have met the necessary conditions. ^15^ Czechia, Hungary and Poland have been EU members for 20 years but still have failed to meet the requirements to join the currency which shows how challenging it can be to align economic status. Georgia, Moldova and Ukraine applied to join the EU in the wake of Russia’s invasion of Ukraine. Before Georgia became a candidate country, they had to address key clarifications whereas Moldova and Ukraine were granted candidate status immediately. From the perspective of a migrant, international migration is more challenging than domestic migration. The introduction of a common labour market has benefited millions of people, however, there are some issues that migrants encounter. One such struggle is a language barrier ( Zalewski, 2021) - there are 24 official languages of the EU with more languages in use in the common labour market such as Icelandic, Norwegian, and regional ones. Having a poor command of the host country’s language can be a barrier to employment or fully integrating into the community. Where a country has a positive attitude towards migrants, they are more likely to integrate ( Naveed & Wang, 2021), however, with the rise of populism in Western Europe in particular, negative attitudes are likely to increase.

## Data Availability

Zenodo: A timeline of freedom of movement in the European Economic Area. https://doi.org/10.5281/zenodo.7225880 ( Barker, 2022). This project contains the following underlying data: - Full list of sources by country Data are available under the terms of the Creative Commons Attribution 4.0 International license (CC-BY 4.0).

## Footnotes

^1^ Switzerland does not participate in the European Economic Area, though agreements are in place that allow access to the single market.

^2^ For example, Spain and Portugal had restrictions lifted earlier. This is explained in more detail later.

^3^ As such, dates of changes are usually mid-year (June) rather than calendar year

^4^ Sources: The Federal Council of Switzerland and The Federal Council of Switzerland. Accessed: 13 March 2024.

^5^ Source: Eurostat.

^6^ The Vatican City operates as part of an association with Italy, with Monaco operating as an associate of France. Andorra and San Marino have reached an Association Agreement (source: The European Commission accessed 14 March 2024.) while Monaco is still to reach the same agreement ( European Commission accessed 14 March 2024). These associations are likened to the EEA agreement.

^7^ Source: S&P Global Market Intelligence Accessed 13 March 2024. There are several economic criteria that countries must fulfil in order to join the Euro Area. Denmark has an opt out and Sweden has no intention to join.

^8^ The EU15 minus the UK.

^9^ The 2023 report on Turkey by The European Commission (2023) noted talks were at a standstill. Accessed 15 March 2024.

^10^ Of the 33 chapters in negotiations (34 for Serbia), Montenegro has the most ’closed’ at 3.

^11^ Source: Reuters. Accessed 28 July 2021.

^12^ Source: Author’s calculations using Eurostat tables nama_10_gdp and demo_pjangroup

^13^ Source: The Guardian. Accessed 14 March 2024.

^14^ Previous separation proposals by the Scottish National Party made when the UK was a member of the EU argued that Scotland would be able to continue EU membership, that is no longer the case.

^15^ Source: European Union, Accessed 16 September 2021

## References

[ref-1] Barker ER : A timeline of freedom of movement in the European Economic Area. *Zenodo.* 2022. 10.5281/zenodo.7225880 PMC10446036 37645342

[ref-2] Barker ER Bijak J : Uncertainty in Migration Scenarios, QuantMig Project Deliverable D9.2.University of Southampton, Southampton,2021.

[ref-3] Benelux Union: Traité instituant l’Union économique Benelux (Treaty of the Benelux Economic Union). Treaty,1958; First accessed 19 July 2021. Reference Source

[ref-4] Cassis I : Report: Free Movement of Workers. Report 1116899, European Free Trade Association,2012. Reference Source

[ref-5] Condinanzi M Lang A Nascimbene B : Citizenship of the Union and Freedom of Movement of Persons.Martinus Nijhoff Publishers, Leiden, The Netherlands,2008. Reference Source

[ref-6] Costa O Schwarzer D Beres P : Sailing on High Seas: Reforming and Enlarging the EU for the 21st Century. Technical report, Franco-German Working Group on EU Institutional Reform,2023. Reference Source

[ref-7] Council of the European Union: EEC Council Regulation 2194/91. Regulation 31991R2194, EUR-Lex. Official Journal: L 206/1, 25.6.1991;1991; First accessed 21 July 2021. Reference Source

[ref-8] Council of the European Union, European Commission: Agreement on the European Economic Area. Treaty, EUR-Lex. Document 31994D0001; Official Journal: OJ L 1, 3.1.1994;1993; First accessed 19 July 2021. Reference Source

[ref-9] European Communities: Treaty of Accession of Denmark, Ireland and the United Kingdom (1972). Treaty 11972B/TXT, EUR-Lex. Official Journal: OJ L 73, 27.3.1972;1972; First accessed 19 July 2021. Reference Source

[ref-10] European Communities: Treaty of Accession of Greece (1979). Treaty 11979H/TXT, EUR-Lex. Official Journal: OJ L 291, 28 May 1979;1979; First accessed 19 July 2021. Reference Source

[ref-11] European Communities: Treaty of Accession of Spain and Portugal (1985). Treaty 11985I/TXT, EUR-Lex. Official Journal: OJ L 302, 15.11.1985;1985; First accessed 19 July 2021. Reference Source

[ref-12] European Communities: Treaty of Accession of Austria, Finland and Sweden (1994). Treaty 11994N/TXT, EUR-Lex. Official Journal: OJ C 241, 29.8.1994;1994; First accessed 19 July 2021. Reference Source

[ref-13] European Communities: Agreement between the European Community and its Member States, of the one part, and the Swiss Confederation, of the other, on the free movement of persons. Treaty 22002A0430(01), EUR-Lex. Official Journal: OJ L 114, 30.4.2002,1999; First accessed 13 March 2024,6–72. Reference Source

[ref-14] European Court: Judgment of the Court (Sixth Chamber) of 27 March 1990. Rush Portuguesa Ldª v Office national d’immigration. Reference for a preliminary ruling: Tribunal administratif de Versailles - France. Act of Accession - Transitional period - Freedom of movement for workers - Freedom to provide services. Case C-113/89. Ruling, EUR-Lex,1991. Reference Source

[ref-15] European Union: Treaty of Accession of the Czech Republic, Estonia, Cyprus, Latvia, Lithuania, Hungary, Malta, Poland, Slovenia and Slovakia (2003). Treaty 12003T/TXT, EUR-Lex. Official Journal: OJ L 236, 23.9.2003;2003; First accessed 19 July 2021. Reference Source

[ref-16] European Union: Treaty of Accession of the Republic of Bulgaria and Romania (2005). Treaty 12005S/TXT, EUR-Lex. Official Journal: OJ L 157, 21.6.2005;2005; First accessed 19 July 2021. Reference Source

[ref-17] European Union: Treaty of Accession of Croatia (2012). Treaty 12012J/TXT, EUR-Lex. Official Journal: OJ L 112, 24.4.2012;2012; First accessed 19 July 2021. Reference Source

[ref-18] European Union: Agreement on the withdrawal of the United Kingdom of Great Britain and Northern Ireland from the European Union and the European Atomic Energy Community. Treaty 02020W/TXT, EUR-Lex. Official Journal: OJ L 029 31.1.2020;2020; First accessed 19 July 2021. Reference Source

[ref-19] Fellmer S : Germany Restricted the Freedom of Movement for Polish Citizens – But Does It Matter? Technical report, EUMAP. FINAL REPORT - COUNTRY CASE STUDIES,2008. Reference Source

[ref-20] Lang IG : Transitional Arrangements in the Enlarged European Union: How Free is the Free Movement of Workers? *Croatian Yearbook of European Law and Policy.* 2008;3(3):241–271. 10.3935/cyelp.03.2007.35

[ref-21] Marshall K : European Economic Community - Free movement of Workers - European Court of Justice Determines That in a Case of Temporary Movement of Workers Member States in Whose Territory the Work is to be Carried Out May Not Impose Conditions Related to the Recruitment of Man-Power or Procurement of Work Permits. Case C-113/89, Rush Portuguesa Lda v. Office national d’immigration, 1990 E.C.R. I-1439, 2 C.M.L.R. 818. Ga J Int’l & Compar L.1991;21:557–575. Reference Source

[ref-22] Nagy SG Šiljak, D : ‘Is the european union still a convergence machine? *Acta Oeconomica.* 2022;72(1):47–63. 10.1556/032.2022.00003

[ref-23] Naveed A Wang C : Can Attitudes Toward Immigrant Explain Social Integration in Europe? EU versus Non-EU Migrant. *Soc Indic Res.* 2021;153:345–383. 10.1007/s11205-020-02492-8

[ref-24] Publications Office of the European Union: Treaty establishing the European Coal and Steel Community. Treaty, EUR-Lex.1951; First accessed 19 July 2021. Reference Source

[ref-25] Rapacki R Prochniak M : EU Membership and Economic Growth: Empirical Evidence for the CEE countries. *The European Journal of Comparative Economics.* 2019;16:3–40. Reference Source

[ref-26] Royo S : Lessons from Spain and Portugal in the European Union after 20 years. *Pôle Sud.* 2007;26:19–45. 10.3917/psud.026.0019

[ref-27] State Secretariat for Migration: Free Movement of Persons Switzerland – EU/EFTA. The Federal Council,2023; Accessed: 13 March 2024. Reference Source

[ref-28] The European Commission: Türkiye 2023 Report , COMMISSION STAFF WORKING DOCUMENT 52023SC0696. The European Union,2023. Reference Source

[ref-29] The six Member States: Belgium, Germany, France, Italy, Luxembourg, Netherlands: Treaty establishing the European Economic Community. Treaty 11957E/TXT, EUR-Lex,1957; First accessed 19 July 2021. Reference Source

[ref-30] Zalewski N : Freedom of movement: Unequal intra-European Union migration due to economic and linguistic barriers. PhD thesis, University of Illinois at Urbana-Champaign.2021.

