# Supplementary material for: A timeline of freedom of movement in the European Economic Area
Source: Open Res Eur. 2024 Mar 21;2:133. Originally published 2022 Dec 6. [Version 3] doi: 10.12688/openreseurope.15042.3 (PMC10446036; doi:10.12688/openreseurope.15042.3)
Supplement: Peer review history

# Reviewer report: Reviewer response for version 3

**DOI:** 10.21956/openreseurope.18942.r39068

**Published:** 2024-04-23

**Authors:** Mario Munta

**Affiliations:** 1 Robert Schuman Centre for Advanced Studies, European University Institute, Fiesole, Toscana, Italy; 2 Faculty of Political Sciences, University of Zagreb, Zagreb, City of Zagreb, Croatia

All outstanding issues have been resolved.

If applicable, is the statistical analysis and its interpretation appropriate?

Not applicable

Is the study design appropriate and is the work technically sound?

Yes

Is the work clearly and accurately presented and does it engage with the current literature?

Yes

Are the conclusions drawn adequately supported by the results?

Yes

Are sufficient details of methods and analysis provided to allow replication by others?

Yes

Are all the source data and materials underlying the results available?

Yes

Reviewer Expertise:

European integration, Economic and Monetary Union, Social Europe, EU employment policy, Europeanisation of Public Policy, European Green Deal

I confirm that I have read this submission and believe that I have an appropriate level of expertise to confirm that it is of an acceptable scientific standard.

# Reviewer report: Reviewer response for version 2

**DOI:** 10.21956/openreseurope.16694.r33353

**Published:** 2023-07-10

**Authors:** Mario Munta

**Affiliations:** 1 Robert Schuman Centre for Advanced Studies, European University Institute, Fiesole, Toscana, Italy; 2 Faculty of Political Sciences, University of Zagreb, Zagreb, City of Zagreb, Croatia

The article “A timeline of freedom of movement in the European Economic Area” written by Emily Barker represents a valuable resource for all scholars interested in the topic of freedom of movement, and particularly labor mobility within the EU/EEA. The empirical contributions in Table 2 and Table 3 can serve as a starting point for qualitative and quantitative research into a variety of topics, including determinants of variation in the duration of labor market access restriction by countries (or country groups), conditions for using reciprocal measures etc. Whilst this version of the manuscript is mostly well-written and sound, I am of the opinion that there are still some minor issues which need to be addressed before this paper becomes the final version. These smaller interventions would improve the manuscript even further.

 - First, a note on this sentence from the intro: ‘’However, joining the Single Market has not always been as simple as joining and assuming equivalent status as Existing Member States (EMS)’’. From this sentence, it is not entirely clear whether the author considers all four freedoms in the single market to be equally difficult to access for new Member States (NMS) or does this only apply to free movement of labor. Please clarify accordingly.

 - in the first sentence of the methodology, the paper claims to conduct a multiple case study. I strongly advise against this claim because it does not reflect the reality and doesn’t do justice to what case study methodology and research look like. Multiple case studies are in-depth, qualitative investigations. I believe you can leave this claim out and refer to document analysis of all countries observed in this paper as a data collection method, and quantitative content analysis as the method of data analysis.

 - related to the previous point, it also seems to me from the description of the methodology and the presented results that the author actually conducted ‘quantitative content analysis’ instead of ‘qualitative content analysis’ as argued in the Analysis section. I suggest the author further looks into the differences between the two methodological approaches, and cites the relevant literature. Given the fact that this article observed documents and coded explicit dates in various documents (accession treaties, safeguard agreements etc.), this approach differs from qualitative content analysis in which latent meanings are extracted from the text to generate categories.

 - the paragraph which discusses the Treaty of Accession leaves out (without an explanation why) a discussion of the 2007 and 2013 enlargement cycles. In the same paragraph, the author forgets to mention that Croatia also applied reciprocal restrictions, which is also evident from Table 2 and Table 3.

 - Given how controversial the topic labor mobility has been in Swiss-EU relations, a broadening of that specific paragraph on p 7/11 is warranted. An explicit reference to the Agreement on the Free Movement of Persons from 2002 is needed. This agreement foresees a gradual opening of the Swiss labor market and a quota system. In the case of Croatia, the European Commission and Croatia repeatedly asked for restrictions to Croatian citizens to be lifted after Croatia’s entry into the EU in 2013, however a ban applied until 2021, only to be reapplied/reintroduced in 2023. Please see for Croatian case: https://www.admin.ch/gov/en/start/documentation/media-releases.msg-id-91561.html. Also, for a general overview: https://www.lexology.com/library/detail.aspx?g=958a571f-06a9-4322-a411-16024aeb0975

 - in Table 2 and Table 3, change acronym for Croatia from HRZ to either HRV, HR or CRO as the internationally recognised versions.

 - please clarify in the note or in the title of Table 2 and Table 3 whether they show the years in which the treaties/agreements foresaw a lifting of restriction, that is, the granting of free movement or do they reflect the actual years when the free movement was granted. Look at the case of Croatia – the table writes that Croatian citizens could access the Swiss labor market only from 2024 – which is inaccurate. Please see previous point for reference. Formally, the Swiss safeguard clause for Croatian nationals can be applied until 2024, however free movement was granted already in 2022. The fact that new quotas were reintroduces for Croatians starting 2023 does not deny the fact that restrictions were lifted at the end of 2021. Please clarify and if needed, include separate figures in brackets for cases where there is a discrepancy in the theoretical and actual revocation of mobility restrictions on the labor market.

 - Figure 1 should be updated to reflect the fact that Croatia entered the Eurozone starting 1 January 2023. Equally, the number of EA countries should be updated to 20. Related to this point, please also correct the numbers in the final section on ‘’Challenges of integration’’ – seven countries have not adopted the euro, and five are still to join (excluding Denmark and Sweden).

If applicable, is the statistical analysis and its interpretation appropriate?

Not applicable

Is the study design appropriate and is the work technically sound?

Yes

Is the work clearly and accurately presented and does it engage with the current literature?

Yes

Are the conclusions drawn adequately supported by the results?

Yes

Are sufficient details of methods and analysis provided to allow replication by others?

Yes

Are all the source data and materials underlying the results available?

Yes

Reviewer Expertise:

European integration, Economic and Monetary Union, Social Europe, EU employment policy, Europeanisation of Public Policy, European Green Deal

I confirm that I have read this submission and believe that I have an appropriate level of expertise to confirm that it is of an acceptable scientific standard, however I have significant reservations, as outlined above.

# Response

**Published:** 2024-03-18

**Authors:** Emily Barker

**Affiliations:** University of Southampton, UK

The author thanks the reviewer for their valuable points and especially the Portuguese example. In the forthcoming new version, I have made changes based on the referee report. Below I have detailed the changes I have made in response to the comments. The sentence “However, joining the Single Market has not always been as simple as joining…” has been amended to focus on free movement of labour. Some caveats apply but it is mainly labour. The term “multiple case study” has been modified to reflect the suggestion. Using the definitions of “The quantitative method seeks to generate a numerical value to either cite prevalence or use in statistical analyses, while the qualitative method seeks to identify a construct or concept within the text using specific words or phrases for substantiation, or to provide a more organized structure to the text being described.” Kleinheksel et al (2020) “Demystifying Content Analysis”),  I agree that quantitative is appropriate. The ToA for 2005 (BGR and ROU) and 2011 (HRV) have been included in the text. The imposition of reciprocal restrictions by Croatia have also been included. The description of tables 2/3 has been updated to reflect the changed situation of the Croatian/Swiss agreement. I have made notes at the end of the text in the “results” section of temporary changes such as Spain. I’ve now included some more details on the Switzerland-Croatia case but for brevity I’ve kept it short. For a similar reason, I’ve kept the table simple as the Swiss-Croatia was the only one that was not completed at the time of the original research. Though, I’ve include a note on the description. I have also included the reference to AFMP agreement. HRZ that appears in tables 2 and 3 has been updated to HRV. The author believes this to be a typo as it is correct in table 1. Figure 1 has been updated accordingly, with notes about future members of the Euro Area and in the paragraph about challenges.

# Reviewer report: Reviewer response for version 2

**DOI:** 10.21956/openreseurope.16694.r33358

**Published:** 2023-07-10

**Authors:** Dion Kramer

**Affiliations:** 1 Vrije Universiteit Amsterdam, Amsterdam, North Holland, The Netherlands

By providing a clear overview of the opening of labour markets to new (EEC/EU/EEA/EFTA) Member States, this article offers a useful addition to the literature on the history of free movement and will be a useful resource for scholarship on free movement and migration. I have three main points for improvement.

 - My main point is a lack of engagement with the (mostly legal) literature pointing at such phenomena as posted work and self-employed work. While workers are typically restricted from entering the labour markets of other Member States during transition agreements, there are typically no restrictions on the free movement of services and establishment. The famous Rush Portuguesa judgment is an example of this: while Portugal was still subjected to transition arrangements, Portuguese companies could already provide services and bring their Portuguese workers to other Member States for that purpose. Same goes for establishment: e.g., Polish citizens could set up a company and start economic activities as self-employed workers in other Member States. In other words: restrictions were merely imposed on the the free movement of workers and not all forms of migration itself (as suggested in the paper). For a comprehensive understanding of (the effects of) transition agreements, the difference between these forms of labour mobility seem pertinent to address. 

 - The discussion/conclusion does not seem to follow necessarily from the findings/results of this paper. One could arguably reflect on the prospect of free movement after (future) accession(s), but could one really reflect on the prospect of accession itself based on the findings? I am not saying that the discussion in its present form is not interesting, but how do your findings specifically add to the wider debate around accession? For one, the Russian war against Ukraine has accelerated the potential and the process of accession, of both Ukraine and other CC and PCCs. Additionally, I struggle to see the relevance of the final subsection on integration in light of the contribution of the article. This brings us to the main point: what is the added value of such a timeline? Perhaps this could be explicated better.

 - Stylistic: There are some typo's and informal use of language ("aren't"). Additionally, I would personally refrain from using abbreviations, especially since there are so many. The article is relatively short and the reader will not benefit from reading abbreviations like CC, PCC, ToA, NMS, EMS, etc.

If applicable, is the statistical analysis and its interpretation appropriate?

Not applicable

Is the study design appropriate and is the work technically sound?

Yes

Is the work clearly and accurately presented and does it engage with the current literature?

Partly

Are the conclusions drawn adequately supported by the results?

Partly

Are sufficient details of methods and analysis provided to allow replication by others?

Yes

Are all the source data and materials underlying the results available?

Yes

Reviewer Expertise:

EU citizenship, free movement of persons, internal market law.

I confirm that I have read this submission and believe that I have an appropriate level of expertise to confirm that it is of an acceptable scientific standard, however I have significant reservations, as outlined above.

# Response

**Published:** 2024-03-18

**Authors:** Emily Barker

**Affiliations:** University of Southampton, UK

The author thanks the reviewer for their valuable points and especially the Portuguese example. In the forthcoming new version, I have made changes based on the referee report. Below I have detailed the changes I have made in response to the comments. I have updated text in the introduction and in “study design” to specify the type of migration restricted. I also referenced the Rush case and referenced the example in Germany of self-employed workers. The original version of this document was written pre the invasion of Ukraine, however, I did update the tables on submission to this journal. The purpose of getting the dates of freedom of movement was outlined in Section 2.  As it was useful to others, I decided to make it a short paper. In light of the new applicant countries and the recognition of Bosnia and Georgia as full candidate countries as well as suggestions from a another reviewer, I have modified the discussion section to include how an assessment of the pros/cons of being in a single market. I have rewritten and tried to make the discussion section follow on from the paper. The relevance of integration is that differences are profound between countries that full integration is not possible. There have been discussions of a fiscal union, in addition to a monetary union, as a method of working towards a more unified stance. If we compare the EU to the US, there are states which are relatively poorer but there is a common language. From an individual’s migration perspective, going from Mississippi to Maryland is not the same as Bulgaria to Germany as a different language is required which does not even use the same alphabet. This level of difficulty makes migration more challenging. I have added details and rephrases to make it more relevant to the paper. A subsection, related to a comment from another review, includes assessing the effectiveness of market membership. I have removed informal words. I have only removed CC and PCC from the main text, though it remains in the table to keep the columns from being unnecessarily wide. ToA has been removed and either specified or referred to explicitly as the Treaty. I haven’t removed EMS and NMS. With the elimination of other acronyms, it might ease the point. NMS and EMS are used several times each.

# Reviewer report: Reviewer response for version 2

**DOI:** 10.21956/openreseurope.16694.r31761

**Published:** 2023-06-20

**Authors:** Francesco Paolo Mongelli

**Affiliations:** 1 Goethe University, Frankfurt, Germany

In my view, the article “A timeline of freedom of movement in the European Economic Area” by Emily Barker provides a very useful support in the discussion and analysis of an indispensable plank of the Single Market: the freedom of mobility within the European Union but also across EFTA and the wider European Economic Area. On one hand, this might support further research into the actual transposition of EU/EEA legislation into national law and the adoption of national decrees to support freedom of movement. On the other hand, it might support research into actual labour mobility across EU/EEA member states. My prior is that freedom of movement might be higher among EU member states, but should still be significant among EEA states: this is an empirical question.

 Major points:

- Would be helpful to clearly distinguish between ratifications, adoption into national law, transposition with help of implementation decrees, enabling provision(s), and so on.
- Concerning Table 2, I am perplexed by the NA: keep blanks instead? Figure 1 is about the EMS (nice figure by the way). 
- In the end implementation of the SM is a matter of degrees and modulation: what might subsequent empirical analysis show in terms of effective labor mobility?
- In the discussion: vague language when mentioning "...align their social, economic and political factors." Which factors?

Minor points:

- The Single Market aims at/promotes....
- It should be “member states”.
- I am not sure that Liechtenstein would appreciate being labelled as an "anomaly", may be unique case?
- Please take out or redraft Footnote 7: not sure UK might appreciate. 

If applicable, is the statistical analysis and its interpretation appropriate?

Partly

Is the study design appropriate and is the work technically sound?

Yes

Is the work clearly and accurately presented and does it engage with the current literature?

Yes

Are the conclusions drawn adequately supported by the results?

Yes

Are sufficient details of methods and analysis provided to allow replication by others?

Partly

Are all the source data and materials underlying the results available?

Yes

Reviewer Expertise:

Economic integration and monetary policy

I confirm that I have read this submission and believe that I have an appropriate level of expertise to confirm that it is of an acceptable scientific standard.

# Response

**Published:** 2024-03-18

**Authors:** Emily Barker

**Affiliations:** University of Southampton, UK

The author thanks the reviewer for his valuable comments on this research.. In the forthcoming new version, I have made changes based on the referee report. Below I have detailed the changes I have made in response to the comments. I have tried to clarify that the dates on these treaties are when they are signed rather than enshrined into law and when the countries actually join rather than it only signed. E.g. Croatia was signed in 2011 but did not join until 2013. NA, was intended for not applicable. On the suggestion of the reviewer. I have updated these to blanks. Thank you for the comment on the figure. The third major point is going beyond the original scope of the paper. However, one significant piece of analysis could be the adjustment of labour market data for which there are significant skill-job mismatches. I have added further details to evaluate the effectiveness or trade offs that occur for joining the common market at the end. I have rephrased factors to status and given examples including democracy, human rights and international cooperation. The factors quotation is one that is essentially a bracket term of the EU itself to cover a wide range of topics in a concise phrase. Aligning these goals is partly why Turkey is made little progress towards becoming a member of the EU but several members have joined the EU since Turkey first approached it, essentially jumping ahead. In the introduction “The Single Market promotes the free movement…” Existing/New Member States changed to existing/new member states. Liechtenstein has been rephrased and updated to include small details on similarly sized micro-states. Footnote 7 and the paragraph has been redrafted. At the time or original writing, the Scottish independence movement was gaining significantly more support, however, after more recent events, support has decreased, especially of the Scottish National Party following their scandals.
